# *Penicillium expansum* Impact and Patulin Accumulation on Conventional and Traditional Apple Cultivars 

**DOI:** 10.3390/toxins13100703

**Published:** 2021-10-04

**Authors:** Ante Lončarić, Bojan Šarkanj, Ana-Marija Gotal, Marija Kovač, Ante Nevistić, Goran Fruk, Martina Skendrović Babojelić, Jurislav Babić, Borislav Miličević, Tihomir Kovač

**Affiliations:** 1 Faculty of Food Technology Osijek, Josip Juraj Strossmayer University of Osijek, Franje Kuhača 20, 31000 Osijek, Croatia; amgotal@ptfos.hr (A.-M.G.); jurislav.babic@ptfos.hr (J.B.); borislav.milicevic@ptfos.hr (B.M.); tihomir.kovac@ptfos.hr (T.K.); 2 Department of Food Technology, University Centre Koprivnica, University North, Trg dr. Žarka Dolinara 1, 48000 Koprivnica, Croatia; bsarkanj@unin.hr; 3 Inspecto Ltd., Industrijska Zona Nemetin, Vukovarska Cesta 239b, 31000 Osijek, Croatia; marija.kovac@inspecto.hr (M.K.); ante.nevistic@inspecto.hr (A.N.); 4 Department of Pomology, Faculty of Agriculture, University of Zagreb, Svetošimunska 25, 10000 Zagreb, Croatia; gfruk@agr.hr (G.F.); mskendrovic@agr.hr (M.S.B.)

**Keywords:** apples, *Penicillium expansum*, mycotoxins, patulin, polyphenols

## Abstract

*Penicillium expansum* is a necrotrophic plant pathogen among the most ubiquitous fungi disseminated worldwide. It causes blue mould rot in apples during storage, transport and sale, threatening human health by secreting patulin, a toxic secondary metabolite that contaminates apples and apple-derived products. Nevertheless, there is still a lack of sufficient data regarding the resistance of different apple cultivars to *P. expansum,* especially ancient ones, which showed to possess certain resistance to plant diseases. In this work, we investigated the polyphenol profile of 12 traditional and 8 conventional apple cultivar and their resistance to *P. expansum* CBS 325.48. Eight polyphenolic compounds were detected; the most prominent were catechin, epicatechin and gallic acid. The highest content of catechin was detected in ‘Apistar’—91.26 mg/100 g of fresh weight (FW), epicatechin in ‘Bobovac’—67.00 mg/100 g of FW, and gallic acid in ‘Bobovac’ and ‘Kraljevčica’—8.35 and 7.40 mg/100 g of FW, respectively. The highest content of patulin was detected in ‘Kraljevčica’ followed by ‘Apistar’—1687 and 1435 µg/kg, respectively. In apple cultivars ‘Brčko’, ‘Adamčica’ and ‘Idared’, patulin was not detected. Furthermore, the patulin content was positively correlated with gallic acid (r = 0.4226; *p* = 0.002), catechin (r = 0.3717; *p* = 0.008) and epicatechin (r = 0.3305; *p* = 0.019). This fact indicates that higher contents of gallic acid, catechin and epicatechin negatively affected and boost patulin concentration in examined apple cultivars. This can be related to the prooxidant activity of polyphenolic compounds and sensitivity of *P. expansum* to the disturbance of oxidative status.

## 1. Introduction

Apples are one of the most popular fruit around the world with an annual production of 87.24 million tons worldwide [1]. The apple is widespread throughout the world because it is cheap, easy to store, available most of the year and it is a nutrient-dense food with many health benefits to consumers [2]. Apples, as well as other fruits and vegetables, are susceptible to different postharvest diseases. Approximately 50% of fruit product loss is caused by different postharvest damage, including decomposition with different pathogens [3]. Susceptibility to infection during storage depends on fruit maturity, in other words, on higher sugar content, water activity decreased firmness, changes in pH, etc. [4]. The most common postharvest pathogen in apples is *Penicillium expansum*. This is manifested in the form of soft rot of the whole fruit, part of the fruit or localized dark spots on the fruit skin and limited tanning of the fruit meat on the cross-section of the fruit. In addition to the reduction of apple quality, secondary metabolism of *P. expansum* produces patulin, a mycotoxin that could lead to acute, subacute and chronic toxic problems, including genotoxicity, immunotoxicity and neurotoxicity [5,6,7,8]. Most often, infection with mycotoxigenic fungi takes place through damaged surfaces, insect wounds or splits that can occur from growing to postharvest storage and markets. After it was classified as mycotoxin, patulin was considered as a measure of quality concerning food safety standards/practices around the world. Therefore, the European Commission has restricted the maximum level of patulin to no more than 50 µg/L in apple juice and apple cider, while in solid apple products and products for infants and young children to 25 µg/kg and 10 µg/kg, respectively [9]. Patulin surveillance in Italy showed that the amount of patulin was 4.77 µg/L and 10.92 µg/L in conventional and organic apple juices, respectively; in Poland 22% of the 754 and in Croatia 21.3% of the 122 apple juice samples had 5 µg/L or more patulin [10,11,12]. Patulin production in apples depends on a whole range of quality factors and interactions between internal and environmental factors such as agrometeorological conditions, geographical area, pathogen load on the fruit, fungal strain and fruit physiological properties [4]. Furthermore, researches have shown that susceptibility to pathogen attack and patulin accumulation in apples also differ between different apple cultivars [13,14,15]. This is because different cultivars differ in their physical and chemical properties, such as hardness, acidity, skin thickness firmness, etc. [16,17,18]. Apple cultivars could be also divided into conventional apple cultivars, the ones that are commercial and we can find in stores, and traditional apple cultivars grown locally in relatively small orchids. Research conducted on four conventional apple cultivars (‘Red Delicious’, ‘Golden Delicious’, ‘Granny Smith’, ‘Fuji’) showed that patulin accumulation is negatively correlated with the acidity of the fruit [19]. Pepeljnjak et al. [20] showed differences in patulin accumulation comparing different conventional apple cultivars ‘Red Delicious’, ‘Golden Supreme’, ‘Gala’, ‘Fuji’, ‘Empire’ and ‘McIntosh’. The ‘Golden Supreme’ (54.2 µg/kg) and ‘McIntosh’ (52.1 µg/kg) showed the highest patulin accumulation among studied apple cultivars [20]. Snini et al. [21], studying 13 different cultivars, showed that patulin is not indispensable in the installation of the disease but acts as a cultivar-dependent aggressiveness factor for *P. expansum*. They strengthened this conclusion by the fact that the addition of patulin to apples infected by the PeΔpatL mutant, lacking one of the genes of the patulin production in the cluster required for patulin synthesis, restored normal *P. expansum* colonization in the apples [21]. The prevention of apple contamination with *P. expansum* in orchards can be achieved by using cultivars that possess greater resistance to diseases. By using such cultivars, ecological and economic benefits in terms of less use of protective agents could also be achieved. Norelli et al. [22] determined the gene responsible for the resistance of the wild apple (*Malus sieversii*) to *P. expansum*. Furthermore, traditional apple cultivars have also shown a certain gene resistance to environmental conditions, plant diseases and other forms of abiotic stress [23]. It has been shown that apples that share close gene profiles show similar responses to a pathogen attack [21,24]. Therefore, it is important to know the gene profile of different apple cultivars. It is one of the factors that shape the characteristics of apple fruits, which determine, among other things, the ability to heal the damage, as well as the sensitivity to blue mould, and consequentially the production of patulin in apples [22]. Research on traditional and commercial apple cultivars has shown that traditional apple cultivars contain higher levels of polyphenols and higher antioxidant activity compared to conventional cultivars [25,26,27]. Polyphenols, molecules with strong antioxidant activity, are synthesized by plants as stress response. Although the mechanism of apple resistance to fungal diseases has not yet been sufficiently investigated, some of the studies have shown that polyphenol compounds are involved in the response to patulin contamination, as they neutralize the free radicals induced by patulin [28]. Sun et al. [29] have shown that cultivars containing higher levels of polyphenols, procyanidins, dihydrochalones, flavonols and phenolic acids are more resistant to infection by *P. expansum* [29]. Currently, there is no available research in the literature on the resistance of Croatian traditional apple cultivars to contamination with *P. expansum*, and consequently on the patulin content in apples. The aims of this study are to examine the resistance of Croatian traditional apple cultivars to infection by P. expansum and accumulation of patulin, and to compare the resistance of traditional and conventional apple cultivars with emphasis on the polyphenol profile of both traditional and conventional apple cultivars.

## 2. Results and Discussion

Among identified polyphenolic compounds (Table 1), catechin and epicatechin were the most abundant polyphenols found in apples. The highest content of catechin was detected in ‘Apistar’ (91.26 mg/100 g of FW) followed by ‘Kraljevčica’ (80.08 mg/100 g of FW). The highest content of epicatechin was detected in ‘Bobovac’ (67.00 mg/100 g of FW) followed by ‘Apistar’ (64.26 mg/100 g of FW). These results are in accordance with the content of individual polyphenols in apples reported in the Phenol-Explorer database [30]. Furthermore, the flavan-3-ols, phenolic acids and dihydrochalcones were determined. Gallic acid was the major phenolic acid detected in investigated apple cultivars. The highest content of gallic acid was detected in apple cultivars ‘Bobovac’ (8.35 mg/100 g of FW) and ‘Kraljevčica’ (7.40 mg/100 g of FW). Regarding dihydrochalcones, phloridzin and phloretin were detected; however, phloridzin was the more prominent dihydrochalcone. Dihydrochalcones are characteristic polyphenolic compounds in apples, where they can represent up to 3% of total polyphenols in apple flesh [31,32]. Results showed that traditional apple cultivars had a higher average content of identified polyphenols. The higher content of individual polyphenols in traditional apple cultivars is in accordance with previously reported results of Jakobek et al. [33], Lončarić et al. [34] and Iacopini et al. [27].

Regarding the antioxidant activity, several methods were proposed to evaluate the antioxidant activity of plant extracts. Since DPPH^•^ assay, based on the inactivation of stable synthetic radicals, is one of the most commonly used in vitro assays and is used for the purpose, Table 2 presents the results of antioxidant activity of ten Croatian traditional and five conventional apple cultivars. The strongest antioxidant activity was observed for the traditional apple cultivars ‘Kraljevčica’ (416.87 mmol TE/L) and ‘Apistar’ (409.63 mmol TE/L). The results are in accordance with the higher content of catechin and epicatechin detected in these cultivars. Such correlation was also reported by Iacopini et al. [27] and is related to the basic structure of the phenols and other structural factors which play a fundamental role in the mechanism by which these compounds are able to scavenge free radicals [35]. Specifically, the antioxidant activity of flavan-3-ols is the result of O-dihydroxy groups in the B-ring, the presence of a C 2–3 double bond in conjunction with 4- oxo in the C-ring, the 3- and 5-hydroxy groups and the 4-oxo function in the A and C-rings which are associated with antioxidant activity [36]. Althrough some studies showed that antioxidant activity is one of the host factors involved in the inhibition of mycotoxin accumulation in apples, it should be emphasized that here the presented results of antioxidant activity may not be the indicator of resistance of particular apple cultivars to *P. expansum* infection.

**Figure 1:**
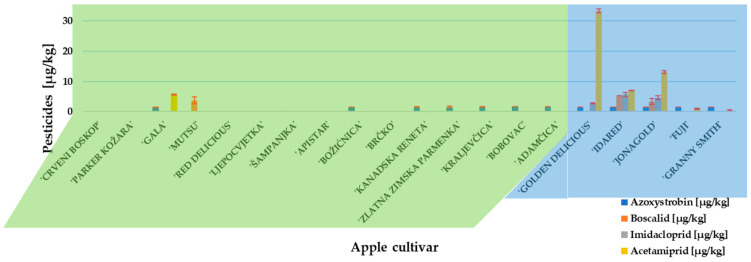
Pesticide content in investigated apple cultivars. Mean ± SD based on three measurements (*n* = 3).

Before approaching an experiment with *P. expansum* inoculation and patulin measurement, the presence and concentration of pesticides residues in traditional and conventional apples cultivars were determined. Two fungicides (azoxystrobin and boscalid) and two insecticides (imidacloprid and acetamiprid) were detected (Figure 1). Azoxystrobin was the only fungicide detected in traditional apple cultivars, while conventional apple cultivars ‘Idared’ and ‘Jonagold’ contained all above-named residues. The insecticide, acetamiprid, was present in the highest concentration (33.36 µg/kg) and detected in ‘Gold Delicious’. However, all detected pesticide concentrations were below the maximum residue level set by the European Commission [37].

**Figure 2:**
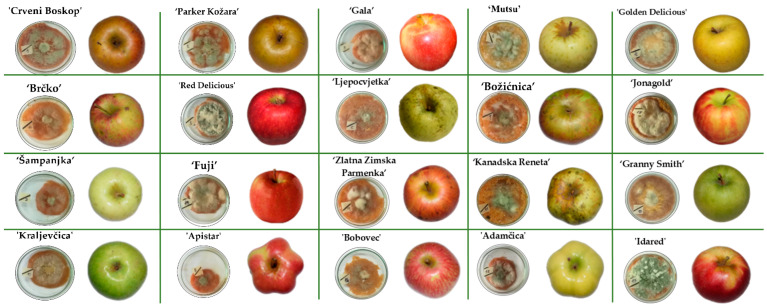
Traditional and commercial Croatian apple varieties after infection with *P. expansum* CBS 325.48 at the end of incubation period when the colonies reached the edge of the apple slice at 29 °C. All incubations were done in triplicate.

The growth of *P. expansum* CBS 325.48 on the apples was monitored and recorded on a 24 h basis until the colony reached the edge of the apple slice. Photographs of the inoculated apples after the end of the incubation period are given in Figure 2. The growth of the *P. expansum* colony depended on the apple cultivar: on average, a shorter growing period of 312 h was observed in conventional apple cultivars, whereas ‘Red Delicious’ had the shortest growing period of 264 h. In traditional apple cultivars, on average, the growing period was 336 h, with ‘Crveni Boskop’ having the longest growing period of 384 h. Several different colonies were observed: a green colony with white margins, a white colony and a colony with green conidia. This observation is in accordance with the report of Tannous et al. [38], although for different *P. expansum* isolate (*P. expansum* NRRL 35695).

The extent of host contamination by *P. expansum* is the result of the interaction between pathogen virulence and fruit resistance. The virulence of *P. expansum* includes viability and pathogenicity, and is closely related to the pathogen and the host itself [39]. In other words, the patulin production will be affected by environmental conditions and quality parameters of the fruit host.

**Figure 3:**
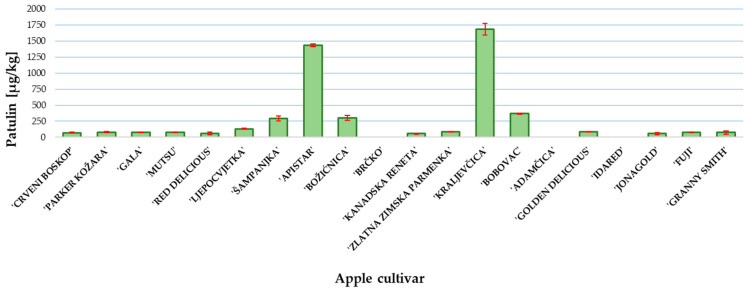
Patulin content in investigated apple cultivars. Mean ± SD based on three measurements (*n* = 3).

The patulin content was determined at the end of the incubation period when the *P. expansum* colony reached the edge of the apple slice. The results of patulin content are presented in Figure 3. The highest content was measured in ‘Kraljevčica’ and was followed by ‘Apistar’ 1687 and 1435 µg/kg, respectively. In all other examined cultivars, except ‘Brčko’, ‘Adamčica’ and ‘Idared’, the patulin concentration was above the regulated level of 25 μg/kg for solid apple products [9]. In the samples of apple cultivars ‘Brčko’, ‘Adamčica’ and ‘Idared’, patulin was not detected (Figure 3).

Since the interplay of pro-oxidant activity of polyphenolic compounds and sensitivity of *P. expansum* cells to the disturbance of oxidative status is expected to mirror into patulin biosynthesis, the correlations between patulin and individual polyphenol compounds detected concentrations are presented in Table 3. The higher content of patulin was positively correlated with higher content of gallic acid (r = 0.4226; *p* = 0.002), catechin (r = 0.3717; *p* = 0.008) and epicatechin (r = 0.3305; *p* = 0.019). Such correlation of results originates from the pro-oxidative effect of flavan-3-ols that provoked reactive oxygen species (ROS) accumulation in *P. expansum* cells, thus triggering the cellular antioxidant defence system (e.g., antioxidant enzymes and/or glutathione synthesis, etc.) and induced patulin biosynthesis, a secondary defence system that lowers ROS levels within the cells [40,41,42]. Antioxidant defence systems can act as ROS scavengers, and they are closely associated with the pathogenicity of *P. expansum* [41]. Patulin is also regulated, among others, by reactive oxygen species (ROS), and undesirable accumulation of intracellular ROS can stimulate patulin production [43]. Furthermore, we detected a relatively small concentration of dihydrochalcones (0.17–2.72 mg/100 g of FW); however, according to some authors, phloridzin could be the major polyphenol responsible for apple resistance to fungal infection. The antifungal activity of phloridzin was explained by the formation of the hydrolyzed product, phloretin, which then oxidizes and forms fungitoxic o-quinone [44,45]. The antifungal activity of phloridzin and its aglycone, phloretin, was previously described [46,47]. Furthermore, Oleszek et al. [48] showed that the fraction of apple extract containing mainly phloridzin in the concentration of 500 μL/mL had strong antifungal activity against different mycotoxigenic fungi.

There is also a possibility of changes in patulin structure as a defence mechanism of apples and formation of masked mycotoxins [49]. This process is well documented for other mycotoxins such as deoxynivalenol and zearalenon [49]; although there is no literature evidence of masked patulin, it was proposed by Saleh and Goktepe [50].

## 3. Conclusion

In conclusion, the results of the study showed considerable insight and comparison of the different traditional Croatian and conventional apple cultivars and their resistance to *P. expansum.* It was shown that traditional apple cultivars were more resistant to infection by *P. expansum* and that, at the same time, they contained higher concentrations of polyphenolic compounds. However, it seems that higher content of polyphenols, particularly flavan-3-ol, induce patulin production. Higher content of gallic acid, catechin and epicatechin boosted the biosynthesized patulin concentration in examined cultivars. That is confirmation of pro-oxidant activity of polyphenolic compounds and sensitivity of *P. expansum* cells to the disturbance of oxidative status. However, as a future perspective, it remains to carry out a broader study with agrometeorological, morphological and physicochemical experiments included with the aim of determination of the resistance of different apple cultivars on *P. expansum* infection and consequent patulin production. This should add to food and feed safety from the patulin occurrence point of view, prevention of economic losses and help to provide sufficient food amounts at a global level.

## 4. Materials and Methods

### 4.1. Chemicals

Folin−Ciocalteu reagent was purchased from Kemika (Zagreb, Croatia); 2,2-diphenyl-1-picrylhydrazyl, catechin (CAS: 154–23–4, ≥99.0%), epicatechin (CAS: 490–46–0; ≥98%), gallic acid (CAS: 149–91–7, ≥97.5), p-cumaric acid (CAS: 501–98–4, ≥98.0%), phloretin (CAS: 60–82–2, ≥99.0%), phloridzin (CAS: 60–81–1, ≥98%), chlorogenic acid (CAS: 327–97–9, ≥95%) and caffeic acid (CAS: 331–39–5, ≥98.0%) were obtained from Sigma Chemical Co. (St. Louis, MO, USA). Methanol (HPLC grade) and orthophosphoric acid (85%) were obtained from Panareac (Barcelona, Spain). Certified standard of patulin and MycoSep^®^ 228 AflaPat columns were obtained from Romer Labs Biopure (Romer Labs, Tulln, Austria). Certified pesticide standard solutions were obtained from CPAchem (CPAchem, Stara Zagora, Bulgaria). LC-MS and HPLC grade acetonitrile was purchased from J.T. Baker (J.T. Baker, Deventer, The Netherlands), while LC-MS grade formic acid and LC-MS ammonium formate were produced by Sigma–Aldrich (Sigma-Aldrich, St. Louis, MO, USA). Ammonium hydroxide solution, ≥25% NH_3_ in H_2_O, was purchased from Honeywell (Offenbach, Germany). Nylon syringe filters, 0.2 µm pore size and 13 mm diameter, were obtained from Agilent (Santa Clara, CA, USA). Ultrapure water was generated by Niro VV system (Nirosta d.o.o., Osijek, Croatia).

### 4.2. Plant Material

**Figure 4:**
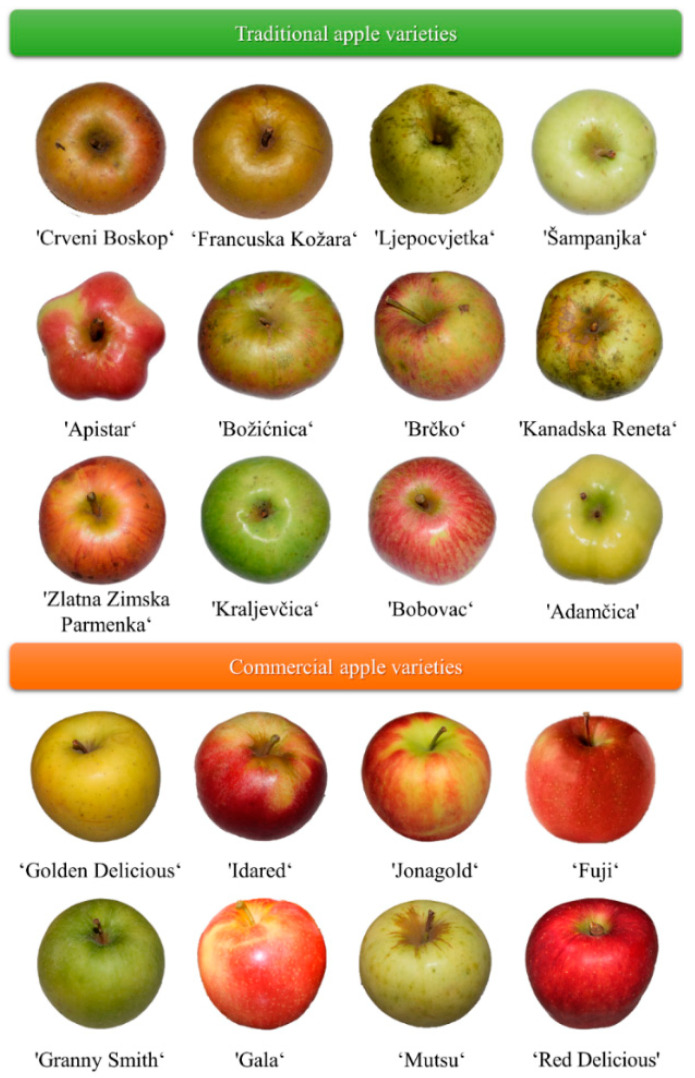
Traditional and commercial apple cultivars used in the experiment [26].

Conventional apple cultivars, ‘Idared’, ‘Jonagold’, ‘Golden Delicious’, ‘Red Delicious’, ‘Granny Smith’ and ‘Mutsu’ were purchased from a local market in the maturity stage from OPG Pavičić, Petrijevci, Osijek, Croatia. The traditional apple cultivars, ‘Crveni Boskop’, ‘Francuska Kožara’ ‘Ljepocvjetka’, ‘Šampanjka’, ‘Apistar’, ‘Brčko’, ‘Bobovec’, ‘Adamčica’, ‘Zlatna Zimska Parmenka’, ‘Božićnica’, ‘Kraljevčica’ and ‘Kanadska Reneta’ were collected from OPG Horvatić, Cvetkovac, 48312 Rasinja, Croatia. All studied apple cultivars (Figure 4) were authenticated by a pomologist [26].

### 4.3. Identification of Polyphenols

Polyphenol identification was performed on a Varian LC system (Agilent, Avondale, PA, USA) equipped with a ProStar 230 solvent delivery module and a ProStar 330 PDA detector. Star Chromatography Workstation software (version 5.52) was used for controlling and quantification of the analysis. Phenolic compound separation was done with an OmniSpher C18 column (250 × 4.6 mm inner diameter, 5 μm, Agilent, USA) protected with a guard column (ChromSep 1 cm × 3 mm, Varian, USA). The mobile phase consisted of the following: solvent A was 0.1% phosphoric acid, and solvent B was 100% HPLC grade methanol. The gradient elution system was: 0−30 min from 5% to 80% B; 30−33 min, 80% B; 33−35 min, from 80% to 5% B; with a flow rate = 0.8 mL/min [51]. A quantity of 5 µL of the sample was injected in duplicate onto the column kept at 50 °C. The chromatograms were monitored in the range of 190 to 600 nm.

### 4.4. Determination of Antiradical Activity

The antioxidant activity (AA) was measured using a DPPH radical according to the methodology described by Brand-Williams [52]. The reaction mixture consisted of 0.2 mL of the extract and 3 mL of DPPH radical solution with 0.5 mM in ethanol. The changes in the color of the radical from deep violet to light yellow were measured at 517 nm using a UV–vis spectrophotometer (Jenway 6300, Bibby Scientific, UK). The antiradical activity (AA) was quantified from the Trolox^®^ calibration curve (50–500 mmol TE/L, R^2^ = 0.9916). The AA was calculated and expressed as millimoles of Trolox^®^ equivalents (TE) per litre of apple extract (mmol TE/L). The measurements were performed in triplicates for each sample.

### 4.5. Determination of the Resistance of the Selected Apple Cultivars to P. expansum

The resistance of the fruits of the selected apple cultivars to *P. expansum* CBS 325.48 was determined by the following method described in Ballseter et al. [53]. For the experiment, the culturing mildew of *P. expansum* CBS 325.48 was prepared on PDA (potato dextrose agar) for 7 days. Apples were sliced on 1 cm thick slices and in the centre of each slice the core was removed using a plug hole puncher with 1 cm diameter, where the discs of a growing culture of *P. expansum* was inserted. Inoculated apple samples were incubated at 29 °C until the *P. expansum* colony reached the edge of the apple slice. Upon reaching the maximum diameter of the colony, the infected apple sample was excluded and stored at −80 °C until it was subjected to the determination of the produced patulin concentration. The fungal growth was measured and pictured every 24 h of growth until mould reached the edge of the apple slice.

### 4.6. Sample Preparation Procedure for Patulin and Pesticide Determination

The sample preparation for the patulin determination followed the MycoSep^®^ 228 AflaPAt procedure. A sample portion of 25 g was extracted by 100 mL solvent mixture (acetonitrile/ultrapure water 84/16, *v/v*) using a mechanical shaker for 30 min. An amount of 8 mL of the raw extract was placed into a test tube and cleaned using MycoSep^®^ column (Romer Labs Division Holding GmgH, Tulln, Austria) by pushing the extract upwards through the column packing material. The cleaned extract was filtered through 0.2 µm nylon filter and injected into a UPLC-MS/MS system. The prepared sample extract was also used for pesticide residues determination. For internal control of procedure, within each sample batch, recovery experiments were conducted by spiking the apple sample with an appropriate amount of patulin and pesticide residues analytical standards, used for the correction of measured concentrations for quantified compounds if recovery was not within the range 90–110% allowed for mycotoxins by Commission Regulation (EC) No 401/2006 [52], and within the range 80–120% allowed for pesticide residues by SANTE/12682/2019 [53].

### 4.7. Patulin and Pesticide Determination

Confirmatory UHPLC-MS/MS analytical methods were used for patulin and pesticide residues determination, both successfully in-house validated and fitted to purpose. For patulin analysis, Waters Acquity H-class UPLC system (Waters, Milford, MA, USA) was employed to perform chromatographic separation using a BEH C18 column (100 × 2.1 mm, 1.7 µm particle size) (Waters, Milford, MA, USA) maintained at 40 °C. Gradient elution was carried out with ultrapure water (eluent A) and acetonitrile (eluent B), both containing 1 mL aqueous solution of ammonium hydroxide, at a constant flow rate of 0.45 mL/min. The injection volume was 20 µL, achieved using an extension loop. The separation started with 100% A, maintained for 1.8 min and followed by a linear decrease to 10% A in 0.5 min with a hold time of 0.7 min, afterwards switching to 100% A and column equilibration to initial conditions in the next 3 min, giving a total run time of 6 min. The UPLC system was coupled to Waters Xevo TQD tandem mass spectrometer (Waters, Milford, MA, USA) equipped with an electrospray ionization interface operating in negative ionization mode. Cone voltage of 18 V and collision energy values (7 V and 11 V, respectively) were optimized for precursor ion of m/z 153, corresponding to deprotonated patulin molecule [M–H]^−^, and product ions of m/z 109 (used for quantification) and m/z 83 (used as a qualifier). The ionization source parameters were as follows: capillary voltage 1.0 kV, extractor voltage 3.0 V, source temperature 150 °C, desolvation temperature 450 °C, cone gas flow 50 L/h and desolvation gas flow 800 L/h (both gases were nitrogen). Collision-induced dissociation was performed using argon as collision gas at a pressure of 4 × 10^−3^ mbar in the collision cell. Instrument control, data acquisition and processing were performed using MassLynx and TargetLynx software (v. 4.1., Waters, Milford, MA, USA). For pesticide residues analysis, instrumental method conditions used were as previously described by Kovač et al. [54].

## Figures and Tables

**Table 1:** The content of individual polyphenol compounds of the investigated apple cultivars.

Apple Cultivar	Gallic Acid	Catechin	Epicatechin	p-Cumaric Acid	Phloridzin	Phloretin	Caffeic Acid	Chlorogenic Acid
	mg/100 g of FW							
**‘Crveni Boskop’**	2.96 ± 0.09 ^k^*	56.55 ± 0.62 ^f^	26.76 ± 0.44 ^f^	3.84 ± 0.01e ^f^	1.44 ± 0.05 ^e^	0.04 ± 0.00 ^d^	0.64 ± 0.01 ^d^	1.28 ± 0.02 ^i^
**‘Parker Kožara’**	2.80 ± 0.07 ^k^	58.85 ± 0.61 ^e^	30.62 ± 0.33 ^e^	0.81 ± 0.08 ^i^	0.45 ± 0.02 ^j^	0.03 ± 0.01 ^e^	0.58 ± 0.05 ^e^	1.89 ± 0.07 ^g^
**‘Ljepocvjetka’**	4.15 ± 0.06 ^g^	18.42 ± 0.12 ^i^	11.22 ± 0.11 ^k^	1.77 ± 0.02 ^h^	2.39 ± 0.03 ^a^	0.33 ± 0.00 ^a^	0.28 ± 0.01 ^i^	3.78 ± 0.02 ^b^
**‘Šampanjka’**	3.14 ± 0.05 ^j^	10.92 ± 0.08 ^m^	7.31 ± 0.05 ^n^	0.66 ± 0.04 ^j^	1.10 ± 0.03 ^g^	0.33 ± 0.01 ^a^	0.68 ± 0.06 ^d^	2.56 ± 0.01 ^e^
**‘Apistar’**	3.99 ± 0.12 ^g^	91.26 ± 0.48 ^a^	64.26 ± 0.86 ^b^	3.79 ± 0.12 ^f^	1.30 ± 0.05 ^f^	<LOD	0.41 ± 0.05 ^h^	1.46 ± 0.20 ^h^
**‘Božićnica’**	4.44 ± 0.06 ^f^	59.85 ± 0.27 ^d^	58.85 ± 0.21 ^c^	6.21 ± 0.04 ^d^	1.76 ± 0.02 ^d^	<LOD	0.25 ± 0.01 ^i^	2.11 ± 0.04 ^f^
**‘Brčko’**	5.66 ± 0.10 ^d^	6.11 ± 0.06 ^o^	17.78 ± 0.11 ^h^	0.47 ± 0.01 ^k^	1.09 ± 0.01 ^g^	<LOD	0.19 ± 0.01 ^j^	0.29 ± 0.06 ^kl^
**‘Kanadska Reneta’**	4.42 ± 0.06 ^f^	59.99 ± 0.13 ^d^	54.04 ± 0.41 ^d^	9.10 ± 0.03 ^a^	1.85 ± 0.01 ^c^	<LOD	0.47 ± 0.03 ^g^	2.61 ± 0.01 ^e^
**‘Zlatna Zimska Parmenka’**	5.15 ± 0.03 ^e^	55.64 ± 0.29 ^g^	53.97 ± 0.19 ^d^	6.81 ± 0.05 ^b^	1.95 ± 0.07 ^b^	<LOD	0.81 ± 0.01 ^b^	3.53 ± 0.04 ^c^
**‘Kraljevčica’**	7.40 ± 0.05 ^b^	80.08 ± 0.28 ^b^	21.51 ± 0.18 ^g^	3.07 ± 0.02 ^g^	0.56 ± 0.05 ^i^	<LOD	0.20 ± 0.00 ^j^	0.24 ± 0.03 ^l^
**‘Bobovac’**	8.35 ± 0.04 ^a^	38.38 ± 0.05 ^h^	67.00 ± 0.76 ^a^	6.42 ± 0.16 ^c^	1.77 ± 0.02 ^d^	0.06 ± 0.01 ^c^	0.76 ± 0.03 ^c^	3.33 ± 0.03 ^d^
**‘Adamčica’**	3.73 ± 0.03 ^h^	15.73 ± 0.77 ^k^	7.99 ± 0.36 ^n^	6.22 ± 0.14 ^d^	0.72 ± 0.05 ^h^	<LOD	1.22 ± 0.04 ^a^	3.95 ± 0.22 ^a^
**’Golden Delicious’**	6.42 ± 0.02 ^c^	3.12 ± 0.07 ^p^	10.13 ± 0.08 ^l^	0.89 ± 0.03 ^i^	0.40 ± 0.02 ^j^	<LOD	0.17 ± 0.01 ^j^	0.38 ± 0.02 ^jk^
**‘Idared’**	5.60 ± 0.30 ^d^	2.32 ± 0.08 ^q^	3.13 ± 0.07 ^q^	<LOD	0.31 ± 0.01 ^k^	<LOD	0.06 ± 0.00 ^kl^	0.47 ± 0.01 ^j^
**‘Jonagold’**	2.86 ± 0.02 ^k^	8.23 ± 0.29 ^c^	16.34 ± 0.81 ^i^	<LOD	0.18 ± 0.01 ^lm^	0.3 ± 0.02 ^b^	0.10 ± 0.00 ^k^	0.04 ± 0.00 ^m^
**‘Fuji’**	1.90 ± 0.03 ^l^	2.88 ± 0.06 ^p^	8.90 ± 0.44 ^m^	<LOD	0.18 ± 0.01 ^lm^	<LOD	0.05 ± 0.00 ^l^	n.d.
**‘Granny Smith’**	4.52 ± 0.25 ^f^	16.71 ± 0.37 ^j^	14.98 ± 0.27 ^j^	<LOD	0.17 ± 0.01 ^m^	<LOD	n.d.	0.07 ± 0.00 ^m^
**‘Gala’**	2.91 ± 0.05 ^k^	8.96 ± 0.16 ^n^	5.91 ± 0.08 ^p^	0.38 ± 0.01 ^k^	0.23 ± 0.00 ^l^	0.02 ± 0.01 ^f^	0.07 ± 0.00 ^kl^	0.06 ± 0.00 ^m^
**‘Mutsu’**	3.75 ± 0.03 ^h^	10.66 ± 0.09 ^m^	6.61 ± 0.02 ^o^	0.49 ± 0.01 ^k^	0.32 ± 0.02 ^k^	0.03 ± 0.02 ^e^	0.05 ± 0.00 ^l^	0.06 ± 0.00 ^m^
**‘Red Delicious’**	3.45 ± 0.01 ^i^	14.67 ± 0.21^l^	8.92 ± 0.00 ^m^	3.95 ± 0.15 ^e^	0.51 ± 0.05 ^i^	0.04 ± 0.00 ^d^	0.52 ± 0.04 ^f^	0.30 ± 0.02 ^kl^

Mean ± SD based on three measured extracts (*n* = 3), * *p* < 0.05. Different letters in each column indicate significant differences at 95% confidence level as obtained by the LSD test. n.d.—not detected; <LOD—limit of detection.

**Table 2:** Antioxidant activity of investigated apple cultivars measured by DPPH assay.

Apple Cultivar	AOA mmol TE/L
**‘Crveni Boskop’**	354.7 ± 9.10 ^j^*
**‘Parker Kožara’**	367.53 ± 2.55 ^hi^
**‘Ljepocvjetka’**	402.37 ± 2.75 ^bc^
**‘Šampanjka’**	376.24 ± 4.67 ^fg^
**‘Apistar’**	409.63 ± 2.93 ^b^
**‘Božićnica’**	407.7 ± 8.08 ^b^
**‘Brčko’**	331.71 ± 1.68 ^kl^
**‘Kanadska Reneta’**	389.79 ± 3.27 ^e^
**‘Zlatna Zimska Parmenka’**	391.97 ± 1.51 ^de^
**‘Kraljevčica’**	416.87 ± 3.02 ^a^
**‘Bobovac’**	368.01 ± 6.17 ^ghi^
**‘Adamčica’**	318.83 ± 2.75 ^m^
**‘Red Delicious’**	370.91 ± 6.26 ^gh^
**‘Idared’**	384.22 ± 1.11 ^ef^
**‘Jonagold’**	398.74 ± 2.75 ^cd^
**‘Fuji’**	375.03 ± 4.42 ^gh^
**‘Granny Smith’**	398.98 ± 3.84 ^cd^
**‘Gala’**	359.54 ± 4.19 ^ij^
**‘Mutsu’**	373.09 ± 0.42 ^gh^
**‘Red Delicious’**	336.07 ± 13.74 ^k^

Mean ± SD based on three measured extracts (*n* = 3), * *p* < 0.05. Different letters in each column indicate significant differences at 95% confidence level as obtained by the LSD test.

**Table 3:** Color map of correlations and *p*-values for correlations of patulin with identified polyphenols.

Polyphenols mg/100 g of FW			Patulin µg/kg								
			r				*p*				
Gallic acid			0.4226				0.002				
Catechin			0.3717				0.008				
Epicatechin			0.3305				0.019				
p-cumaric acid			0.1588				0.271				
Phloridzin			0.0612				0.673				
Phloretin			−0.1727				0.230				
Caffeic acid			−0.0031				0.983				
Chlorogenic acid			−0.0491				0.735				
r >=	−1	−0.80	−0.60	−0.40	−0.20	0	0.20	0.40	0.60	0.80	1
*p* <=	0.001		0.010	0.025	0.050	0.100	0.150	0.200	0.350	0.500	1

## Data Availability

The data presented in this study are available on request from the corresponding author.

## References

[1] FAOSTAT FAOSTAT: Statistical Database. http://www.fao.org/faostat/en/#data/QCL.

[2] Le Gall S., Even S., Lahaye M. (2016). Fast Estimation of Dietary Fiber Content in Apple. J. Agric. Food Chem..

[3] Carmona-Hernandez S., Reyes-Pérez J.J., Chiquito-Contreras R.G., Rincon-Enriquez G., Cerdan-Cabrera C.R., Hernandez-Montiel L.G. (2019). Biocontrol of postharvest fruit fungal diseases by bacterial antagonists: A review. Agronomy.

[4] Kumar D., Barad S., Sionov E., Keller N.P., Prusky D.B. (2017). Does the host contribute to modulation of mycotoxin production by fruit pathogens?. Toxins.

[5] Zhai Q., Gong X., Wang C., Zhao J., Zhang H., Tian F., Chen W. (2019). Food-borne patulin toxicity is related to gut barrier disruption and can be prevented by docosahexaenoic acid and probiotic supplementation. Food Funct..

[6] Pal S., Singh N., Ansari K.M. (2017). Toxicological effects of patulin mycotoxin on the mammalian system: An overview. Toxicol. Res..

[7] Kovač T., Šarkanj B., Borišev I., Djordjevic A., Jović D., Lončarić A., Babić J., Jozinović A., Krska T., Gangl J. (2020). Fullerol C60(OH)24 Nanoparticles Affect Secondary Metabolite Profile of Important Foodborne Mycotoxigenic Fungi In Vitro. Toxins.

[8] Kovač M., Šubarić D., Bulaić M., Kovač T., Šarkanj B. (2018). Yesterday masked, today modified; what do mycotoxins bring next?. Arch. Ind. Hyg. Toxicol..

[9] The European Parliament and the Council of the European Union Commission Regulation (EC) (2006). No 1881/2006, Maximum levels for certain contaminants in foodstuffs. Off. J. Eur. Union.

[10] Szymczyk K., Szteke B., Goszcz H. (2004). Patulin content in Polish apple juices [Wystepowanie patuliny w krajowych sokach jabukowych.]. Rocz. Panstw. Zakl. Hig..

[11] Spadaro D., Ciavorella A., Frati S., Garibaldi A., Gullino M.L. (2007). Incidence and level of patulin contamination in pure and mixed apple juices marketed in Italy. Food Control.

[12] HAH (2017). Croatian Food Agency—Scientific Report About Patulin in Apple Juice.

[13] McCallum J.L., Tsao R., Zhou T. (2002). Factors affecting patulin production by Penicillium expansum. J. Food Prot..

[14] Jackson L.S., Beacham-Bowden T., Keller S.E., Adhikari C., Taylor K.T., Chirtel S.J., Merker R.I. (2003). Apple quality, storage, and washing treatments affect patulin levels in apple cider. J. Food Prot..

[15] Zhong L., Carere J., Lu Z., Lu F., Zhou T. (2018). Patulin in apples and apple-based food products: The burdens and the mitigation strategies. Toxins.

[16] Chávez R.A.S., Peniche R.Á.M., Medrano S.A., Muñoz L.S., Ortíz M.d.S.C., Espasa N.T., Sanchis R.T. (2014). Effect of maturity stage, ripening time, harvest year and fruit characteristics on the susceptibility to Penicillium expansum link of apple genotypes from Queretaro, Mexico. Sci. Hortic..

[17] Jurick W.M., Janisiewicz W.J., Saftner R.A., Vico I., Gaskins V.L., Park E., Forsline P.L., Fazio G., Conway W.S. (2011). Identification of wild apple germplasm (Malus spp.) accessions with resistance to the postharvest decay pathogens Penicillium expansum and Colletotrichum acutatum. Plant Breed..

[18] Nybom H., Ahmadi-Afzadi M., Rumpunen K., Tahir I. (2020). Review of the impact of apple fruit ripening, texture and chemical contents on genetically determined susceptibility to storage rots. Plants.

[19] Marín S., Morales H., Hasan H.A.H., Ramos A.J., Sanchis V. (2006). Patulin distribution in Fuji and Golden apples contaminated with Penicillium expansum. Food Addit. Contam..

[20] Pepeljnjak S., Šegvić M., Ožegović L. (2002). Citrininotoxinogenicity of Penicillium spp. isolated from decaying apples. Braz. J. Microbiol..

[21] Snini S.P., Tannous J., Heuillard P., Bailly S., Lippi Y., Zehraoui E., Barreau C., Oswald I.P., Puel O. (2016). Patulin is a cultivar-dependent aggressiveness factor favouring the colonization of apples by Penicillium expansum. Mol. Plant Pathol..

[22] Norelli J.L., Wisniewski M., Fazio G., Burchard E., Gutierrez B., Levin E., Droby S. (2017). Genotyping-by-sequencing markers facilitate the identification of quantitative trait loci controlling resistance to Penicillium expansum in Malus sieversii. PLoS ONE.

[23] Fischer M., Fischer C. (2004). Genetic resources as basis for new resistant apple cultivars. J. Fruit Ornam. Plant Res..

[24] Janisiewicz W.J., Saftner R.A., Conway W.S., Forsline P.L. (2008). Preliminary evaluation of apple germplasm from Kazakhstan for resistance to postharvest blue mold in fruit caused by Penicillium expansum. HortScience.

[25] Jakobek L., Barron A.R. (2016). Ancient apple varieties from Croatia as a source of bioactive polyphenolic compounds. J. Food Compos. Anal..

[26] Lončarić A., Skendrović Babojelić M., Kovač T., Šarkanj B. (2019). Pomological Properties and Polyphenol Content of Conventional and Traditional Apple Cultivars from Croatia. Pomol. Prop. Polyphen. Content Conv. Tradit. Apple Cultiv. Croat..

[27] Iacopini P., Camangi F., Stefani A., Sebastiani L. (2010). Antiradical potential of ancient Italian apple varieties of Malus×domestica Borkh. In a peroxynitrite-induced oxidative process. J. Food Compos. Anal..

[28] Ahmadi-Afzadi M., Nybom H., Ekholm A., Tahir I., Rumpunen K. (2015). Biochemical contents of apple peel and flesh affect level of partial resistance to blue mold. Postharvest Biol. Technol..

[29] Sun J., Janisiewicz W.J., Nichols B., Jurick W.M., Chen P. (2017). Composition of phenolic compounds in wild apple with multiple resistance mechanisms against postharvest blue mold decay. Postharvest Biol. Technol..

[30] Neveu V., Perez-Jiménez J., Vos F., Crespy V., du Chaffaut L., Mennen L., Knox C., Eisner R., Cruz J., Wishart D. (2010). Phenol-Explorer: An online comprehensive database on polyphenol contents in foods. Database.

[31] Jakobek L., Krivak P., Medvidović Kosanović M., Šter A., Jukić A. Dihydrochalcones in old apple varieties from Croatia. Proceedings of the International Conference 16th Ružička days “TODAY SCIENCE—TOMORROW INDUSTRY”.

[32] Guyot S., Marnet N., Laraba D., Sanoner P., Drilleau J.F. (1998). Reversed-Phase HPLC following Thiolysis for Quantitative Estimation and Characterization of the Four Main Classes of Phenolic Compounds in Different Tissue Zones of a French Cider Apple Variety (Malus domestica Var. Kermerrien). J. Agric. Food Chem..

[33] Jakobek L., Ištuk J., Buljeta I., Voća S., Žlabur J.Š., Babojelić M.S. (2020). Traditional, Indigenous Apple Varieties, a Fruit with Potential for Beneficial Effects: Their Quality Traits and Bioactive Polyphenol Contents. Foods.

[34] Lončarić A., Matanović K., Ferrer P., Kovač T., Šarkanj B., Babojelić M.S., Lores M. (2020). Peel of traditional apple varieties as a great source of bioactive compounds: Extraction by micro-matrix solid-phase dispersion. Foods.

[35] Sadeghipour M., Terreux R., Phipps J. (2005). Flavonoids and tyrosine nitration: Structure–activity relationship correlation with enthalpy of formation. Toxicol. Vitr..

[36] López M. (2003). Study of phenolic compounds as natural antioxidants by a fluorescence method. Talanta.

[37] European Commission. https://ec.europa.eu/food/plant/pesticides/eu-pesticides-database/products/?event=details&p=23.

[38] Tannous J., Atoui A., El Khoury A., Francis Z., Oswald I.P., Puel O., Lteif R. (2016). A study on the physicochemical parameters for Penicillium expansum growth and patulin production: Effect of temperature, pH, and water activity. Food Sci. Nutr..

[39] Yu L., Qiao N., Zhao J., Zhang H., Tian F., Zhai Q., Chen W. (2020). Postharvest control of Penicillium expansum in fruits: A review. Food Biosci..

[40] Musial C., Kuban-Jankowska A., Gorska-Ponikowska M. (2020). Beneficial properties of green tea catechins. Int. J. Mol. Sci..

[41] Li B., Chen Y., Zhang Z., Qin G., Chen T., Tian S. (2020). Molecular basis and regulation of pathogenicity and patulin biosynthesis in Penicillium expansum. Compr. Rev. Food Sci. Food Saf..

[42] Finotti E., Parroni A., Zaccaria M., Domin M., Momeni B., Fanelli C., Reverberi M. (2021). Aflatoxins are natural scavengers of reactive oxygen species. Sci. Rep..

[43] Delgado J., Ballester A.-R., Núñez F., González-Candelas L. (2019). Evaluation of the activity of the antifungal PgAFP protein and its producer mould against Penicillium spp postharvest pathogens of citrus and pome fruits. Food Microbiol..

[44] Gessler C., Patocchi A., Sansavini S., Tartarini S., Gianfranceschi L. (2006). Venturia inaequalis Resistance in Apple. CRC. Crit. Rev. Plant Sci..

[45] Gosch C., Halbwirth H., Stich K. (2010). Phloridzin: Biosynthesis, distribution and physiological relevance in plants. Phytochemistry.

[46] Baldisserotto A., Malisardi G., Scalambra E., Andreotti E., Romagnoli C., Vicentini C., Manfredini S., Vertuani S. (2012). Synthesis, Antioxidant and Antimicrobial Activity of a New Phloridzin Derivative for Dermo-Cosmetic Applications. Molecules.

[47] Shim S.-H., Jo S.-J., Kim J.-C., Choi G.-J. (2010). Control Efficacy of Phloretin Isolated from Apple Fruits Against Several Plant Diseases. Plant Pathol. J..

[48] Oleszek M., Pecio Ł., Kozachok S., Lachowska-Filipiuk Ż., Oszust K., Frąc M. (2019). Phytochemicals of apple pomace as prospect bio-fungicide agents against mycotoxigenic fungal species—in vitro experiments. Toxins.

[49] Loncaric A., Dugalic K., Mihaljevic I., Jakobek L., Pilizota V. (2014). Effects of sugar addition on total polyphenol content and antioxidant activity of frozen and freeze-dried apple Purée. J. Agric. Food Chem..

[50] Brand-Williams W., Cuvelier M.E., Berset C. (1995). Use of a free radical method to evaluate antioxidant activity. LWT Food Sci. Technol..

[51] Ballester A.R., Norelli J., Burchard E., Abdelfattah A., Levin E., González-Candelas L., Droby S., Wisniewski M. (2017). Transcriptomic response of resistant (Pi613981–malus sieversii) and susceptible (“royal gala”) genotypes of apple to blue mold (penicillium expansum) infection. Front. Plant Sci..

[52] European Commission (2006). Commission Regulation (EC) No 401/2006 of 23 February 2006 laying down the methods of sampling and analysis for the official control of the levels of mycotoxins in foodstuffs. Off. J. Eur. Union.

[53] Guidance SANTE (2019). Guidance Document on Analytical Quality Control and Method Validation Procedures for Pesticides Residues Analysis in Food and Feed. https://www.eurl-pesticides.eu/userfiles/file/EurlALL/AqcGuidance_SANTE_2019_12682.pdf.

[54] Kovač M., Bulaić M., Jakovljević J., Nevistić A., Rot T., Kovač T., Dodlek Šarkanj I., Šarkanj B. (2021). Mycotoxins, Pesticide Residues, and Heavy Metals Analysis of Croatian Cereals. Microorganisms.

